# Combining six genome scan methods to detect candidate genes to salinity in the Mediterranean striped red mullet (*Mullus surmuletus*)

**DOI:** 10.1186/s12864-018-4579-z

**Published:** 2018-03-27

**Authors:** Alicia Dalongeville, Laura Benestan, David Mouillot, Stephane Lobreaux, Stéphanie Manel

**Affiliations:** 1 CEFE UMR 5175, EPHE, PSL Research University, CNRS, UM, SupAgro, IRD, INRA, 34293 Montpellier, France; 2 MARBEC UMR 9190, CNRS – IRD – Université Montpellier – Ifremer, 34095 Montpellier, France; 3 Departement de Biologie, Institut de Biologie Intégrative et des Systèmes (IBIS), Université Laval, Québec, Canada; 4 Laboratoire d’Ecologie Alpine, UMR-CNRS 5553, Université Joseph Fourier, BP53 38041 Grenoble, France

**Keywords:** Adaptive genomics, Genome scan, Candidate genes, Mediterranean Sea, *Mullus surmuletus*, Salinity

## Abstract

**Background:**

Adaptive genomics may help predicting how a species will respond to future environmental changes. Genomic signatures of local adaptation in marine organisms are often driven by environmental selective agents impacting the physiology of organisms. With one of the highest salinity level, the Mediterranean Sea provides an excellent model to investigate adaptive genomic divergence underlying salinity adaptation. In the present study, we combined six genome scan methods to detect potential genomic signal of selection in the striped red mullet (*Mullus surmuletus*) populations distributed across a wide salinity gradient. We then blasted these outlier sequences on published fish genomic resources in order to identify relevant potential candidate genes for salinity adaptation in this species.

**Results:**

Altogether, the six genome scan methods found 173 outliers out of 1153 SNPs. Using a blast approach, we discovered four candidate SNPs belonging to three genes potentially implicated in adaptation of *M. surmuletus* to salinity. The allele frequency at one of these SNPs significantly increases with salinity independently from the effect of longitude. The gene associated to this SNP, *SOCS2*, encodes for an inhibitor of cytokine and has previously been shown to be expressed under osmotic pressure in other marine organisms. Additionally, our results showed that genome scan methods not correcting for spatial structure can still be an efficient strategy to detect potential footprints of selection, when the spatial and environmental variation are confounded, and then, correcting for spatial structure in a second step represents a conservative method.

**Conclusion:**

The present outcomes bring evidences of potential genomic footprint of selection, which suggest an adaptive response of *M. surmuletus* to salinity conditions in the Mediterranean Sea. Additional genomic data such as sequencing of a full-genome and transcriptome analyses of gene expression would provide new insights regarding the possibility that some striped red mullet populations are locally adapted to their saline environment.

**Electronic supplementary material:**

The online version of this article (10.1186/s12864-018-4579-z) contains supplementary material, which is available to authorized users.

## Background

Adaptive genomics aims to understand the molecular basis of local adaptation in species experiencing a wide range of environmental gradients. This emerging research field may help predicting how a species will respond to future environmental changes [1] as well as delineating sustainable management units [2–4]. This important area of research has taken advantage of the arrival of next generation sequencing (NGS) with reduced sequencing costs, allowing thousands of markers, both potentially neutral and adaptive, to be sequenced in hundreds of individuals [5]. These genome-scale sets of markers open the door to identify potential targets of natural selection (i.e., loci showing divergent patterns of allele distribution linked to selective pressures) in model and non-model species [6, 7], and a large variety of genome scan tools are now available [8].

The field of adaptive genomics has extensively studied terrestrial organisms comparatively to marine ones, potentially owing to the lack of available reference genomes for marine species [9]. Yet, marine habitats are rapidly changing and understanding how species will face these environmental modifications in the coming years is a major concern [10]. In this context of improving genomic information to help better predict species response to global warming, adaptive genomic studies has already investigated in the marine realm considering different environmental features such as temperature [11, 12], salinity [13, 14] or even bathymetry [15]. More particularly, salinity is expected to be a selective agent driving local adaptation of several teleost fishes [16, 17] since adaptation to specific osmotic conditions involves molecular, physiological or behavioral changes. Recent work on European bass [14], Atlantic cod [18] and three-spine stickleback [19, 20] have already reported a suite of single nucleotide polymorphism (SNP) within or closely located to genes involved in osmoregulation, altogether leading to a wide list of targeted salinity and osmoregulation genes known for teleost fishes (reviewed in Dennenmoser et al. [17]).

Detecting signals of selection linked to salinity variation may benefit the recent advances in the field. Indeed, adaptive genomics is still a fast evolving study field, with the recent development of numerous analytical methods [8]. These methods are based on different statistical models and assumptions regarding neutral population structure, and then may give results that are not congruent [21, 22]. The majority of the adaptive genomic studies in wild populations usually focuses on few of them [17, 23–25], and comparisons of various methods on empirical datasets are still lacking [8], whereas there were evidences of such need [12, 26].

**Fig. 1:**
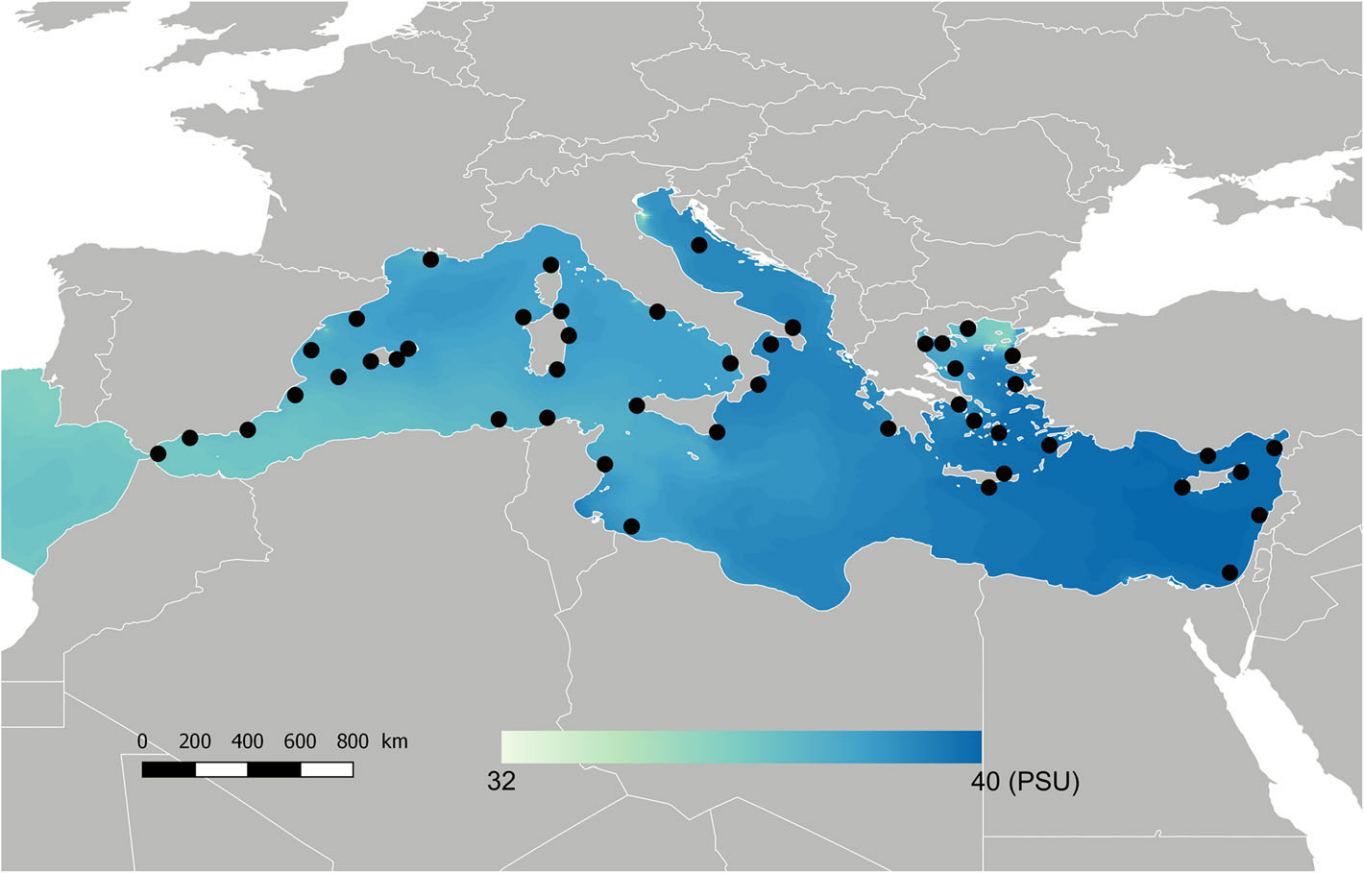
Map of the mean annual Sea Surface Salinity (SSS_max_), averaged from 1990 to 2013, in the Mediterranean Sea. The black dots indicate the position of the 47 study sites

In the present study, we investigated the potential for local adaptation to salinity in an exploited marine fish species, the striped red mullet (*Mullus surmuletus*) along the Mediterranean Sea, using 1153 SNP markers on 47 locations. Due to its enclosed geography and high evaporation rate, the Eastern Mediterranean basin displays high levels of salinity compared to the Western basin and the Northeastern Atlantic [27] (Fig. 1). In this particular area, we tested the hypothesis that salinity may act as a selective agent for *M. surmuletus* populations*.* A previous seascape genetics study using the same extensive marine spatial database showed no genetic differentiation in separated populations, but a longitudinal pattern of isolation by distance [28]. In this study, part of the wide neutral genetic variation was explained by the sea surface salinity variable, suggesting that *M. surmuletus* populations are locally adapted to this environmental variable. In the view of increasing our capacity to detect genomic signatures of selection, we combined six commonly used genome scan methods. Unsurprisingly, we observed inconsistency among the six genome scan methods and we further interpreted their outcomes regarding the algorithms and statistical model behind each of them. Then, we blasted the totality of outlier sequences found (i.e., those detected by at least one genome scan method) on published available fish genomic resources. We were then able to identify four candidate genes potentially involved in salinity tolerance of the striped red mullet.

## Methods

### Species, area and sampling design

The striped red mullet (*Mullus surmuletus*) is a demersal fish species distributed in the Northeastern Atlantic Ocean, from the British Isles in the North to Senegal in the South. This species inhabits coastal areas from 0 to 100 m depth, and has high commercial value in the Mediterranean Sea. The study area covers the whole Mediterranean coastline, including islands. Our sampling design consisted of 47 sites distributed along the whole range of this species across the Mediterranean Sea (Fig. 1). A total of 727 adults of *M. surmuletus* were sampled between April and November 2014. Specimens were obtained from small-scale fisheries landings at each site. Fish samples consisted of fin clips of pectoral and caudal fins conserved in 96% ethanol prior to storage at 4 °C.

### Genetic data and SNP calling

Extraction of genomic DNA was undertaken using the DNeasy Blood & Tissue Kit (Qiagen) according to the manufacturer’s protocol. DNA quality was assessed by running 3 μL of each DNA sample on 1% agarose gels. DNA concentration was determined using NanoDrop 8000. We individually genotyped 541 fish samples from these 47 sites using a genotyping by sequencing approach [28]. Six 96-plex GBS libraries were constructed using restriction enzyme ApeKI (recognition site: GCWGC) following a protocol modified from Elshire et al. [29], and sequenced at the Institute of Genomic Diversity at Cornell University using the Illumina HiSeq 2500 (100 bp, single-end reads). Each library was sequenced on a separate HiSeq flowcell lane.

Raw read sequences were filtered according to quality base calling, removing sequences with average Phred quality below 25. Trimming was performed to remove low quality bases at the extremities of the reads (Phred quality below 20). Sequences shorter than 60 bp were discarded from the dataset. For each sequenced library, the number of raw reads and filtered data are provided in Additional file 1: Table S1 in Supporting Information. SNP calling was performed using the Tassel 3.0 Universal Network Enabled Analysis Kit (UNEAK [30]). The GBS procedure produced individual sequences with a very low coverage, which lead to a high number of missing genotypes in the dataset at the individual level. The Stacks software [31] has also been used and showed similarly prohibitive missing data for individual genotype calling. To overcome this bias, we considered pooling together the individual samples belonging to the same site, in order to accurately estimate allele frequency. Indeed, pooled and individually determined allele frequencies are expected to be similar [32]. This ‘pooling strategy’ allowed us to produce a dataset of 47 ‘pools’ (i.e., sampling sites) containing between nine and eighteen individuals, and whose sequence coverage was >10X and showing a minor allele frequency > 0.05 in all the dataset. The final dataset contained the allele frequencies of 47 pools at 1153 SNPs. The parameters used in both UNEAK and Stacks are detailed in Additional file 1: Appendix S1.

### Population structure

Genetic differentiation between the pairs of 47 sites was quantified by the Wright’s pairwise F_ST_, calculated using the R package ‘polysat’ [33]. To test for Isolation-by-Distance (IBD), we performed a Mantel test between pairwise F_ST,_ computed from allele frequencies and marine geographical distances, computed as least-cost path distances with infinite resistance values assigned to landmasses. We also performed a Principal coordinates analysis (PCoA) of the 47 sites using the Nei genetic distance calculated from SNPs allele frequencies to analyse the population structure. In order to test for isolation by resistance, we used Mantel tests between genetic differentiation (pairwise F_ST_) and environmental distances (maximum Sea Surface Temperature and Salinity). For both environmental variables separately, we calculated pairwise environmental distances as the difference in temperature/salinity between every pair of locations.

### Sea surface salinity

The Mediterranean Sea is an evaporation basin where the evaporation rate exceeds the precipitations [34]. The inflow through the Strait of Gibraltar balances the freshwater loss, which result in a gradient of increasing salinity from west to east [35, 36]. In this study, we aimed to test whether the maximum daily Sea Surface Salinity (SSS_max_) may be a potential agent of divergent selection in *M. surmuletus.* This environmental variable has been identified as a driver of the wide neutral genetic variation of *M. surmuletus* suggesting isolation by adaptation [28]. In the Mediterranean Sea, salinity and temperature are strongly correlated (Pearson’s r^2^ = 0.55; pvalue < 0.001), which makes it difficult to disentangle the adaptive effect of one or the other. Because *M. surmuletus* has a wide distribution in the North-East Atlantic, which encompasses a large temperature gradient compared to the gradient within the Mediterranean Sea, we expect salinity to be the main driver of adaptation here. Hence, we focused our analyses on salinity.

SSS_max_ was computed by NEMOMED8 [37], which has a resolution of 1/8° [38] from the period 1990–2013. The daily data were averaged over the whole period to infer the mean SSS_max_.

### Genome scan methods for detecting the signal of selection

Environmental association (EA) approaches detect SNPs that are significantly correlated to environmental variables. The null hypothesis (H0) is that genetic variation is only due to limited dispersal and genetic drift [39], thus there is no correlation between SNP allele frequencies and the environmental variable (e.g.*,* SSS_max_). We describe below the five EA methods that we used to detect candidate SNPs (Table 1).

**Table 1:** Description of the genome scan methods used to detect candidate outlier SNPs

Method	R package	Categories	Correction for spatial or population structure	Data	# outliers detected (# unique)	Reference
Linear regression (LM)	LM	Linear model	No	Allele frequencies	129 (78)	R development Core Team
Redundancy Analysis (RDA)	vegan	Multivariate method	Yes	Allele frequencies	11 (0)	Legendre & Legendre 2012 [73]
Generalized linear spatial mixed models (gINLAnd)	gINLAnd	Mixed model	Yes	Read counts	7 (0)	Guillot et al. 2013 [49]
Latent factor mixed models (LFMMs)	LEA	Mixed model	Yes	Read counts	0 -	Frichot et al. 2013 [50]
Moran spectral outlier detection (MSOD)	PCNM and adespatial	Multivariate method	Yes	Allele frequencies	7 (1)	Wagner et al. 2017 [43]
Principal Component Analysis (PCadapt)	pcadapt	Multivariate method	No	Allele frequencies	88 (43)	Duforet-Frebourg et al. 2015 [74]

#### Linear regression (LR)

Linear regression (LM) is the simplest way to test for significant correlations among SNP allele frequencies and environmental variables assuming that allele frequencies follow a normal error model, and vary linearly with the environmental variables [40]. Here significance of correlations among allele frequencies in each pool (=site) and SSS_max_ was considered when both the chi-squared test and the t-test (H0, no correlation among allele frequencies and SSS_max_) were significant as recommended by Joost et al. [41].

#### Redundancy analysis (RDA)

RDA is an ordination method extending linear regression to multivariate response data (i.e.*,* allele frequencies of multiple SNPs). First, linear regressions are computed between allele frequencies in each site and explanatory environmental variables at each SNP. Then fitted values of those regressions are analysed simultaneously using a principal component analysis (PCA) to produce ordination axes that are linear combinations of the original explanatory variables. This allows accounting for the multivariate properties of SNPs. We performed RDA to test the effect of SSS_max_ on allele frequencies in each pool (site). We also used latitude and longitude of the sampling sites as explanatory variables to control for the effect of potential neutral spatial structure. Outlier SNPs were identified on each of the first three ordination axes as SNPs with a ‘locus score’ that was ±3 SD from the mean score for that axis [22]. We then calculated the correlation between the allele frequency at each outlier and SSS_max_. We considered the correlation to be significant at a *p*-value lower than 0.05. The RDA was performed using the R package ‘*vegan*’ [42].

#### Moran spectral outlier detection (MSOD)

MSOD uses Moran eigenvector maps (MEM) to quantify the distribution of allele frequencies across a range of spatial scales represented by MEM spatial eigenvectors [43]. This procedure follows two steps: *i*) First, the power spectrum of each SNP is compared to the average power spectrum of all of the SNPs in order to identify outliers that show an unusual power spectrum. The power spectrum of a SNP is the squared correlation coefficient of that SNP with the MEM eigenvectors. *ii*) The second step uses Moran spectral randomization [44] to test the association between the outlier SNPs detected at the first step and an environmental variable (here SSS_max_), while accounting for spatial autocorrelation between sites.

MEM provides a spectral decomposition of the spatial relationships among the study sites [45]. A set of 46 MEM axes were computed from the geographic coordinates of our 47 sampling sites using the R package ‘*PCNM*’ version 2.1–4 [46]. The power spectrum of each SNP corresponds to the vector of squared correlations between its allele frequencies in each site and every MEM axes. Deviation of SNPs from the average power spectrum was measured using z-scored, and a list of candidates was obtained with a cut-off of 0.05.

At step 2, Moran Spectral Randomization (MSR) uses a randomization approach to build a null hypothesis of no correlation between SSS_max_ and allele frequencies at each SNP detected as an outlier at step 1, given the power spectra of the candidate outliers. We performed MSR using the R package ‘*adepsatial*’ version 0.0–7 with 199 permutations [47]. The final set of candidate outliers was obtained as the SNPs significantly correlated to SSS_max_ under a threshold *p*-value of 0.05.

#### Spatial generalized linear mixed models (gINLAnd)

The software gINLAnd aims to quantify the correlation between allele counts at each site and the environmental variation using a logistic regression accounting for spatial autocorrelation between samples sites due to population history. For each SNP, gINLAnd fits a generalized linear spatial mixed model using an estimation of spatial covariance between sites as random factor, and the environmental variable as fixed explanatory variable. Spatial covariance is estimated according to the Integrated Nested Laplace Approximation (INLA) method proposed by Rue et al. [48].

For computational optimization, the parameters of the spatial covariance, *τ* and *κ*, were calculated on a random subset of 500 SNPs and then taking the average values. For each SNP, we then fitted a full model including the environmental (SSS_max_) and the random (*τ* and *κ*) variables, and a reduced model including the random variables only. Analyses were performed using the function *ginland.inferences* of the gINLAnd R package [49]. The SNP-environment association was deemed significant when the full model had a higher likelihood than the reduced model, evaluated with Bayes factor (BF). We used a conservative threshold of BF > 3 (log(BF) > 1.1) to consider the association as significant.

#### Latent factor mixed model (LFMM)

Latent factor mixed models (LFMM) are mixed linear models that test for the correlations between allele counts and one environmental variable (here SSS_max_), accounting for the neutral structure through latent factors [50]. Parameters are estimated in a Bayesian context. LFMM was run with 10,000 burning sweeps and 20,000 effective sweeps of the Gibbs sampling algorithm. We ran ten replicates of the analysis and combined the z-scores over replicates following the recommendations described in [51]. First, we took the median z-score over the ten replicates, and calculated the genomic inflation factor, λ. Then, we calculated the adjusted *p*-values using λ. These adjusted p-values were used to decide on the significance of the association. We obtained lists of candidate SNPs using the Benjamini–Hochberg procedure under false discovery rates ranging from 0.05 to 0.2.

The number of latent factors (unobserved variables) has to be specified by the user for the analysis. Here, we used K = 1 and K = 2 to correct for population structure since multivariate analyses showed no clustering but isolation by distance (IBD; Additional file 1: Figure S1). We used the implementation of LFMM in the R software using the ‘*LEA*’ package version 1.6.0 [51] to conduct the analysis.

#### Principal component analysis (PCA) with PCAdapt

In addition to these five EA methods, we used a population differentiation (PD) method, which investigates the existence of highly differentiated SNPs across the genome without assuming the effect of a particular environmental variable. PCAdapt estimates population differentiation at each SNP and compares the value with an expected estimation, calculated from the genome wide background. The null assumption tested is that each SNP is not under selection.

When using pooled data, PCAdapt first generates individual genotypes at every SNPs based on the allele frequencies of the pools, using a binomial model. For each pool, one hundred individuals were generated, as advised in the tutorial. We checked the adequacy between allele frequencies estimated from these individuals and the allele frequencies of the pools, and as expected, both were strongly correlated (mean Pearson’s correlation coefficient overall SNPs = 0.96).

PCAdapt uses a PCA, a multivariate analysis able to detect population structure from the genetic data. The software constructs a set of axes from the previously generated individual genotypes, which are linear combination of the initial variables (Principal Components, PCs). The PCs are produced on a criterion of maximal variance. PCAdapt extends this analysis by calculating correlations between SNPs and a set of retained PCs [52]. Candidate SNPs are detected as the ones that correlate significantly to this set of PCs under a specified False Discovery Rate (FDR). The analysis was performed using the version 3.0.4 of the R package ‘*PCAdapt*’ [52]. We first assessed the optimal K value (i.e., optimal number of genetic groups), from 1 to 20, using a screen plot of the proportion of variance explained by each PC using the *PCAdapt* function. We then retained K = 2 and calculated the FDR of the pvalues associated with Mahalanobis distance estimated by PCAdapt, using the *qvalue* function of the R package ‘*qvalue*’ [53]. Finally, we obtained a list of candidate SNPs under an expected FDR α = 0.05, meaning that 5% of the candidate SNPs are expected to be false positives.

### Blasting outlier sequences

We assessed whether any of the 173 candidate SNPs identified by the six EA and PD approaches belong to known annotated genes in the NCBI’s nr database [54]. We blasted all candidate sequences of 80 bp length against the available NCBI genomic resources for teleost, cartilaginous and bony fishes. We retained only the sequences showing a minimal homology of at least 90% and a minimal alignment length of 15 bp, with a E-value of 10^− 4^. We then investigated the function of these genes according to the UNIPROT database (http://www.uniprot.org). To ascertain whether a given mutation was synonymous or non-synonymous, the codon containing the SNP variants was translated into an amino acid according to the location of the start codon. From the subset of candidate genes found in the NCBI’s nr database, we selected the genes that were already known to have an influence in salinity adaptation in other organisms.

### Using partial linear regression to correct for spatial genetic patterns in SNP candidates

Longitude was previously suggested to have a wide influence on all the genomic variation of this species [28], and is strongly correlated to salinity. In order to test whether salinity was still significantly correlated to MAF variation independently of the effect of longitude, we performed a partial linear regression between the minor allele frequencies (MAF) at each location and the salinity gradient observed, for each candidate SNP retained by genome scans methods that do not correct for spatial structure (LM and PCAdapt). To perform this partial linear regression framework, we used the *rda* function available in the ‘*vegan*’ package and we tested the significance of the relationship using 1000 permutations.

## Results

### SNP dataset

Individual genotyping produced 626,867 SNPs. Filtering for an individual average minimal coverage of 5X and an average maximal coverage of 10× kept 1491 SNPs. Thirteen individuals with less than 3000 reads were discarded. Pooling individuals sampled in the same site allowed producing 1153 SNPs for 47 sites, with coverage of 10X and 100% of the sites genotyped at each marker (i.e. no missing data). On average over every loci, 11 individuals contribute to each pool, with a median of 10 and standard deviation of 2.91. The distribution of the MAF per SNPs was computed to verify the validity of SNPs calling and filtering. This distribution is shown in Additional file 1: Figure S2. The number of individuals for each site is shown in details in Additional file 1: Table S3.

### Genetic structure

Pairwise F_ST_ ranged from 0.018 to 0.065, with a mean of 0.033, showing weak genetic differentiation between sites, as expected for a mobile marine species. The PCoA showed differentiation between Gibraltar (site 20) and all the other sites (Additional file 1: Figure S1). The Alboran Sea (sites 3, 4, and 5) also appeared slightly differentiated from the rest of the Mediterranean Sea (Additional file 1: Figure S1). The Mantel test between pairwise F_ST_ and marine geographical least-cost distances was significant (r_M_ = 0.30, *p*-value < 0.001), suggesting a pattern of isolation-by-distance in the data. Concerning isolation by resistance, the Mantel test between F_ST_ and pairwise distances in salinity was significant (r_M_ = 0.28, pval < 0.001), whereas it was not significant for temperature (p-value = 0.09). This suggests that salinity may influence *M. surmuletus* genetic structure in the Mediterranean Sea.

### Detection of candidate outliers

**Fig. 2:**
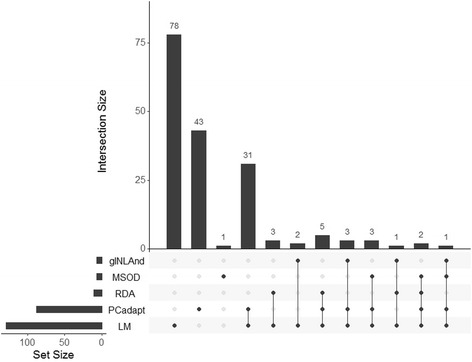
UpSet diagram: matrix layout for all intersections of five genome scan methods (i.e., LM, PCAdapt, RDA, MSOD, gINLand) sorted by their number of outliers detected and the uniqueness of the method. LFMM is not represented since it did not identify any outlier (SNP) Dark circles in the matrix indicate the methods that are part of the intersection. Vertical bars show the size of the intersection (i.e., number of shared outliers). Intersections of a single method (the first three vertical bars) represent the set of unique outliers detected only by that method and no other one. The size of each set (i.e., the total number of outlier detected by each method) is displayed by the horizontal bars on the left, and provided in Table 1

Overall, the five EA and one PD approaches detected a total of 173 SNPs (15%) putatively under divergent selection across all locations, whereas the intersection of all methods did not found any. To identify SNPs detected as outliers by several methods, we delineated subsets of unique and shared number of candidate SNPs (hereafter outliers) detected by this set of approaches (Fig. 2). Results were highly variable in terms of number and identity of the outliers, which were drastically different depending on the method used. Overall, LM and PCAdapt detected the largest sets of outliers, with respectively 129 and 88 candidate SNPs. Other methods such as gINLAnd, MSOD and RDA detected widely smaller sets, ranging from seven (gINLAnd and MSOD) to eleven (RDA). LFMM found no outlier under any of the thresholds and the latent factors tested (K = 1 or K = 2).

Among the methods detecting the largest sets of outliers, LM and PCAdapt, shared 45 common SNPs (35% and 51% of their respective sets), and they both also identified large unique subsets of outliers, with respectively 78 (60%) and 43 (49%) outliers that were not uncovered in any other methods (Fig. 2). Except one outlier that was only detected using MSOD approach, 100% and 70% of the outliers found by gINLAnd, MSOD and RDA were also pinpointed by LM and PCAdapt respectively (Fig. 2).

### Gene ontology of candidate genes

The BLAST search of these 173 outlier sequences against the NCBI’nr yielded to a set of 131 outlier sequences successfully identified as belonging to known genes in all NR database, using permissive criteria. Yet, only 30 outlier sequences were kept according to their homology, alignment length and their E-value (see criterion described in Methods). Among these 30 sequences, all were found in teleost and bony fishes, and 11 in cartilaginous fishes, as expected. From these 30 successfully annotated genes, four were already known to potentially play a role in adaptation to salinity. The sequences of the SNP *TP106031*, *TP221669*, *TP346795* and *TP61263* were located into these four genes (Table 2). Out of these four candidate SNPs, only the SNP *TP106031*, detected by PCAdapt, was non-synonymous. This SNP belongs to *MSRA* gene, which encodes the methionine sulfoxide reductase, a protein already known to be involved in salt tolerance processes in barley *Hordeum vulgare* [55]*.* The location of the mutation tends to increase the rate of reaction of MSRA when the variant attaches a lipid [56]. This may provide an advantage in correcting the oxidation state of cells due to the increase in salinity, but also maybe due to an increase in temperature or oxygen levels. The SNP *TP221669*, also identified as outlier by PCAdapt, is located in the *CYP7A1* gene, which encodes cholesterol 7-alpha-monooxygenase, a protein involved in bile acid and bile salt metabolism [57]. Finally, we found the SNPs *TP346795*, identified by LM, and the SNP *TP61263*, detected by both LM and PCAdapt, which belong to the same gene: *SOCS2.* This gene produces proteins that are inhibitors of cytokine signaling pathways and key physiological regulators of immune system in vertebrates [58]. The transcription of this gene was suggested to be tightly linked to salinity variation in marine species, as previously demonstrated by De Zoysa et al. [59] in the disk abalone (*Haliotis discus discus*) and by Komoroske et al. [60] in the delta smelt (*Hypomesus transpacificus*).

**Table 2:** Characterization of high-quality BLAST matches obtained in comparison of striped red mullet genotype by sequencing SNP against NCBI database. We only retained SNPs located in genes with putative functions that are compatible with the hypothesis of salinity adaptive selection acting on encoded protein. R^2^ and *P*-value referred to the linear regression between SSS_max_ and minor allele frequencies of each SNP

SNP	Detection method	Uniprot	Gene	Protein	General function	R^2^	*P*-value
TP221669	PCadapt	AIT82984	CYP7A1	Cholesterol 7-alpha-monooxygenase	Bile acid and bile salt metabolism, bile salt export pump	0.045	0.079
TP106031	PCadapt	ACO09386	MSRA	Peptide methionine sulfoxide reductase	Enzymatic restriction of methionine sulfoxyde to methionine	0.043	0.081
TP346795	LM	ACV85622	SOCS2	Suppressor of cytokine signaling 2	Involved in JAK-STAT signaling cascades	0.009	0.218
TP61263	PCadapt, LM	ACV85622				0.17	0.002

**Fig. 3:**
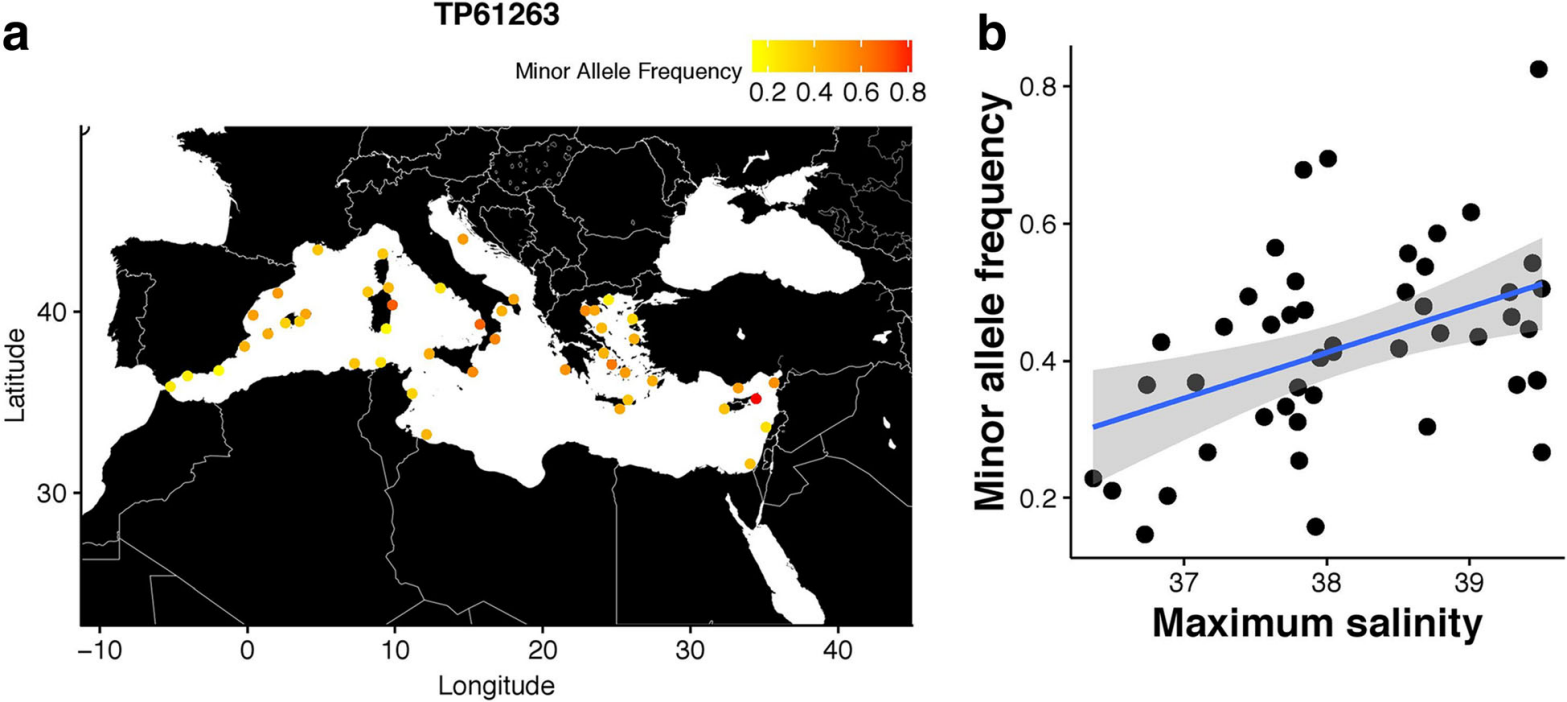
**a** Map showing the minor allele frequency of the SNP *TP61263* at each of our 47 study sites. **b** Correlation between minor allele frequency of SNP *TP61263* and maximum annual sea surface salinity (SSS_max_), including loess smoothing function and confidence interval (grey area). Partial linear regression showed that SSS_max_ still explained 6.8% of the variation at this SNP after longitude influence was removed

When analyzing each candidate SNP separately, we demonstrated that only one SNP, *TP61263*, was still significantly correlated to SSS_max_ when spatial distribution was taken into account (partial linear regression). Indeed, we found that longitude and SSS_max_ were both significant but the variance explained by SSS_max_ (17.1%) was twice the one explained by longitude (8.9%). In addition, SSS_max_ still explained 6.8% of the variation at this SNP after longitude influence was removed (Fig. 3b). Revealing correlation between the minor allele frequency of this candidate SNP along with the salinity gradient (SSS_max_) confirmed that the SNP *TP61263,* may be a potential candidate for the local adaptation of the striped red mullet to salinity in the Mediterranean Sea (Fig. 3a and b).

Considering that salinity and temperature are correlated in the Mediterranean Sea, we assumed that the candidate SNPs we identified could also be associated with variations in temperature. So, we tested the correlation between the allele frequencies of candidate SNPs (173 SNPs identified as outliers) and maximum temperature computed by NEMOMED8 [37]. The absolute value of Pearson’s correlation coefficient varied between 0.005 and 0.438 (mean = 0.178). The correlation was significant for four SNPs (*p*-value < 0.005), but none of the previous ones identified as belonging to known genes by the BLAST. We can then conclude that salinity seems to be the main driver influencing genetic variation observed at the candidate SNP detected.

## Discussion

The most salient findings of this study are that, combining the six genome scan methods and the blast of the sequences containing the candidate SNPs against fish genomes, lead to the identification of four potential candidate genes. Those four genes harbor metabolism functions that may be involved in adaptation to salinity in the striped red mullet populations, and other marine species since there were also previously described as potential gene candidates. Additionally*,* we underlined that using a various set of genome scan methods may facilitate uncovering potential genomic footprints of selection, since this strategy help to reduce the number of false negatives. In the following, we described how our work might enhance our understanding of salinity adaptation processes in marine species and its importance for evolution of such species facing climate change. Furthermore, we discussed methodological issues when environment is correlated to spatial variation.

### Functional genomics: Uncovering potential gene candidates for adaptation to salinity

Spatial variations in salinity are expected to induce local adaptation of marine populations [61, 62], and genes implicated in salinity tolerance have been identified in various marine organisms by both experimental and empirical studies. For example, local adaptation to salinity has been demonstrated for the Baltic Sea three-spine sticklebacks (*Gasterosteus aculeatus*) using common garden experiments [63]. Genes potentially implicated in osmoregulation have been identified in model marine species such as the European sea bass [14] and the three-spine sticklebacks [19, 20] using genome sequencing combined with genome scan, transcriptome analyses and QTL approaches. On the Atlantic cod, a study of Berg et al. [18] combining genome scan and landscape genomics detected genomic regions under directional selection associated with differences of salinity in the Baltic and the North Sea.

The Mediterranean mean annual salinity varies between about 32 to 40 PSU, which makes it one of the saltiest seas on earth [27]. Thus, adaptation of Mediterranean fish species to these stressing osmotic conditions is expected. Evidence of adaptive genetic structure in the eastern part of the Mediterranean Sea has been shown in the peacock wrasse (*Symphodus tinca*) using genome scan, although it was not clearly associated with any environmental factor [64]. Another recent work from Ruggeri et al. [65] demonstrated an association between outliers from microsatellite genetic data and environmental factors (salinity, oxygenation and temperature) in the European anchovy (*Engraulis encrasicolus*). This pattern was then further investigated by Catanese et al. [66] who used genomic and transcriptomic data to reveal that low salinity associated with river mouths may influence local adaptation processes equally in the Tyrrhenian Sea and North Adriatic Sea but they found no outlier loci with gene function clearly related to salinity variations. Here, we performed a blast research to accurately identify known genes underpinning adaptation though a gene ontology approach [5] on our exhaustive list of potential candidate sequences. As expected, genome scan analyses detected a vast majority of outlier SNPs that were not located within any gene since these SNPs could be linked to a selected gene, or implicated in gene regulation, or simply be false positives [67]. Yet, four outlier SNPs belonging to three relevant potential candidate genes were found by our blast search. For instance, we discovered the candidate gene *CYP7A1*, which encodes the cholesterol 7 alpha-monooxygenase, an enzyme initiating the classic and alternative bile salt pathways [57]. More particularly, this gene was 8000-fold more transcriptionally expressed in the liver of mature (i.e., lamprey living in a saline environment) male sea lamprey over immature (i.e., lamprey living in a non-saline environment) male adults [68]. On the other hand, we also found the *MSRA* gene, as being a potential gene candidate for salinity adaptation since its repair function has been shown to protect cells from oxydative damage [69]. Indeed, the MSRA gene produces the methionine sulfoxide reductase A, an antioxidant protein that was upregulated in a salt-sensitive genotype of barley (*Hordeum vulgare*), suggesting that MSRA may be involved in plant adaptation to salt stress [55]. Yet, the most interesting candidate was the *SOCS2* gene that is part of the suppressors of cytokine signaling (*SOCS*) gene family. *SOCS* genes are implicated in regulation of a variety of signal transduction pathways, which are involved in immunity, growth and development of organisms [58]. Eight *SOCS* genes have been identified, among which *SOCS2* has been described as a multifunctional protein that playing roles in signaling pathways and development of central nervous system [70, 71]. Komoroske et al. [60] have shown that *SOCS2* was expressed under osmotic stress conditions in a marine fish species, the delta smelt; and Martinez Barrio et al. [72] identified it as a potential gene candidate for euryhaline adaptation in the Baltic herring (see Supplementary file 3A of their study). Similarly, De Zoysa et al. [59] highlighted its implication in osmotic tolerance in a gastropod species, the disk abalone (*Haliotis discus discus*).

Detecting outlier markers showing minor allele frequencies linked to environmental variables is a first step towards the identification of adaptive processes and demonstrating the existence of such correlation does not prove that local adaptation occurs in the wild. Then, outcomes from population genomic studies should be further investigated at a functional level, to test the functional importance of the candidate SNP in a specific ecological context. For that purpose, additional genomic data such as sequencing of a full-genome and transcriptome analyses of gene expression would be required to provide new insights on the possibility that this gene is really involved in local adaptation process of *M. surmuletus* populations in the Mediterranean Sea. Nevertheless, the entire list of candidate genes found here may serve future studies as a useful database to investigate local adaptation process in species experiencing changes in salinity.

### Comparing approaches for detecting genomic signal of selection

The outcomes from the six genome scan methods varied widely in term of the number and identity of outliers detected in this study (Fig. 2). Such inconsistencies between methods were expected since their algorithms and prior assumptions considerably differ. All methods, except gINLAnd and PCAdapt, assume a linear relationship between SNP allele frequencies and the environmental variable [43, 50, 73]. gINLAnd uses a logit transformation of the allele frequencies [49], whereas PCAdapt maximizes their variances in a PCA without accounting for any environmental variable [74].

Correcting for confounding effects, such as spatial structure or allele surfing, was recently at the heart of genome scan methods development [26]. Therefore, one major difference among the genome scan methods performed in this study was whether this method integrates population or spatial structure correction or not. Indeed, as expected, methods that do not account for population or spatial structure (PCAdapt and LM) detected the largest sets of outliers, whereas gINLAnd, MSOD and RDA, which accounts for it, identified fewer candidates (between seven and eleven). Controlling for spatial structure or IBD is expected to decrease the number of false positive, but also to reduce the power to detect true adaptive SNPs, thus limiting the ability of the methods to detect genomic signals of selection [75]. Such a control normally allows reducing the False Discovery Rate (FDR), by increasing the number of true positives relative to the total number of positives. This strategy is especially relevant when demographic patterns such as isolation by distance or allele surfing [76] may mimic genomic signals of selection along an environmental gradient, which is the case of our dataset since longitude is strongly correlated to salinity (i.e., SSS_max;_ *r* = 0.85, *p*-value = 5.14 × 10^− 14^; Fig. 3a). Thus, the set of outliers detected by PCAdapt and LM is expected to contain a high rate of false positives, but probably also includes more true positives [26]. Therefore, using methods that do not correct for population or spatial structure may represent an efficient strategy to uncover potential candidate genes overall when environmental gradient is confounded with spatial structure. The function of these genes can then be verified first using a blast search and in a second step with a more direct approach such mutagenesis, shedding light on the adaptation process, from genotype to phenotype.

### Limits of the study

The relatively low number of markers (1153 SNPs) used in our study is expected to only represent a small fraction of the genome. Thus, our adaptive genomic study based on these SNPs most likely leaves out genes possibly involved in salinity adaptation but which do not contain - or are not linked to - any SNPs in this dataset [77]. In addition, the union of six genome scans methods identified 15% of the SNPs as outliers, which is an extremely high proportion and surely contains a high number of false positives. However, the aim of this study is to provide a large set of outliers, and then test their relevance though the observation of their minor allele frequency distribution and using blast alignments. Hence, a high false discovery rate is not prohibitive here, since the candidate SNPs were not directly considered as adaptive and as we underlined the need to further investigate their impact at the phenotype level. In that case, using the combination of well-calibrated genome scan methods is an efficient strategy to increase our ability to detect any potential genomic footprint of selection as it has also been suggested by François et al. [26].

Candidate SNPs detected by genome scan are not direct evidence of local adaptation, as explicitly described in Benestan et al. [12]. Therefore, these candidate SNPs identified from the combination of these methods with BLAST and correlative approaches, are only preliminary evidences comforting the hypothesis of an adaptive response of *M. surmuletus* to the osmotic conditions in the Mediterranean Sea. Furthermore, confounding environmental variables such as habitat, pH, water quality or selective harvesting may have induced selective pressure similar to salinity and testing the influence of such variables would also be relevant in order to accurately define local adaptation to salinity [16]. Yet, the strong correlation between salinity and other environmental features, such as longitude and temperature makes the identification of loci only responding to salinity challenging.

## Conclusion

Several studies investigated fish species response to salinity in the Baltic and North Sea [18, 73–75], and only few were focusing on the Mediterranean Sea (but see the studies of Ruggeri et al. [65] and Catenese et al. [66]). Our results bring evidences in favor of the hypothesis of an adaptive response of the striped red mullet to Mediterranean salinity, and produced an exhaustive list of potential candidate genes. We also highlighted the interest of combining different genome scan methods since using the intersection of several methods, as advised in de Villemereuil et al. [78], decreases the amount of false positives but using the union limits the risk of missing a true selected locus [12]. Choosing a combination of genome scan methods obeys to a compromise between limiting type I and type II errors (i.e., reducing the FDR while maximizing the power of the analysis). Hence, this decision will strongly depend on the objectives of the studies, but also on the dataset (e.g., pool-seq or individual genotypes, number of genotyped markers) and a priori knowledge (i.e., is there any population structure or IBD? is the environmental variable correlated to space or other variables?).

In the current context of climate changes, adaptation of marine organisms to temperature is of greatest concern [3]. Yet, salinity is an important variable to consider, since its level is also expected to increase in the future due to reduced precipitations and higher evaporation rates [36, 79]. Identifying genes associated with key environmental factors, such as salinity and temperature, will help assessing the relative importance of evolutionary adaptation, and thus provide insight on species adaptive potential to the environmental modifications predicted to result from global change [3]. Conservation strategies could benefit from this new adaptive genomic knowledge, which could be used to implant evolutionary thinking in decision frameworks [80, 81]. Combining evolutionary adaptation with consideration of species and habitat representation and connectivity into conservation goals will support the aim to reduce the impacts of climate change on biodiversity [3, 82].

## Additional files

Additional file 1: **Appendix S1.** Supplementary methods: sequence filtering and SNPs calling. **Fig. S1.** PCoA of the 47 sites computed using the Nei genetic distance. **Fig. S2.** Histogram showing the distribution of MAF per SNPs for the 47 sites. **Table S1.** Number of raw reads and filtered data for each sequenced library. **Table S2.** Parameters used in SNPs calling with UNEAK and Stacks. **Table S3.** Additional information on the sampling sites. (DOCX 284 kb)
