# Supplementary material for: Combining six genome scan methods to detect candidate genes to salinity in the Mediterranean striped red mullet (Mullus surmuletus)
Source: BMC Genomics. 2018 Mar 27;19:217. doi: 10.1186/s12864-018-4579-z (PMC5870821; doi:10.1186/s12864-018-4579-z)
Supplement: Supplementary file 1 — Appendix S1. Supplementary methods: sequence filtering and SNPs calling. Fig. S1. PCoA of the 47 sites computed using the Nei genetic distance. Fig. S2. Histogram showing the distribution of MAF per SNPs for the 47 sites. Table S1. Number of raw reads and filtered data for each sequenced library. Table S2. Parameters used in SNPs calling with UNEAK and Stacks. Table S3. Additional information on the sampling sites. (DOCX 284 kb) [file 12864_2018_4579_MOESM1_ESM.docx]

**Additional file 1**

**Appendix S1. Supplementary methods: sequence filtering and SNPs calling**

*SNPs calling using UNEAK*

SNP calling was performed using the Tassel 3.0 Universal Network Enabled Analysis Kit (UNEAK; Lu *et al.*, 2013). UNEAK is a non-reference GBS SNP calling pipeline that has been developed as an extension of the Java program TASSEL (Bradbury *et al.*, 2007). UNEAK proceeds in three steps: (1) the reads are cut to 64bp; (2) these sequences are gathered in tags (*i.e.* identical sequences) for each individual sample; (3) The tags are aligned by pair with a single mismatch. In Uneak plugin UMergeTaxaTagCount, the maximum tag number was set to 10^9^ and the minimum tag for a count to 5 (options –m and –c respectively).

The output file describing the coverage of the alleles for each detected SNP in each individual was used for further filtering of the data. SNPs were first filtered individually to get loci with the best coverage and significant reliability in SNP calling: (a) a minimal mean coverage of 5 among the individuals, (b) a maximal mean coverage of 10 among the individuals, to discard repetitive sequences. The maximum coverage threshold was defined according to distribution of individual coverage in this experiment (mean = 3.11 ± 3.56). Further filtering was performed to remove individuals with an insufficient number of sequences (< 3000 reads). For each SNP, we calculated the ratio of individuals for which the coverage was 0 or 1, and removed SNPs from the dataset when this ratio was higher than 10%.

As the individual coverage of the sequencing was low, we pooled individuals collected in the same site (9 to 18 individuals per pool) to get a more reliable coverage. The reads corresponding to the loci that passed the filters detailed in the previous paragraph were gathered for every individuals in each pool. SNPs were re-filtered to keep a minimal coverage of 10 in each pool with no missing data, which lead to a total of 1153 SNPs. Then, allele counts and allele frequencies per pool have been computed for each of the 1153 SNPs. The filters used for SNPs calling are summarized in Table S2.

*SNPs calling using Stacks*

In order to test whether a different algorithm would improve the dataset, SNPs calling has also been performed with Stacks (Catchen *et al.*, 2011), using the scripts developed in *Stacks workflow* (Normandeau, 2016). Reads were truncated to 80pb, then grouped in tags using the *ustacks* unit with a maximum of three mismatches between sequences (M=3), since an important variability is expected in an abundant fish species such as *M. surmuletus*; and a minimum coverage of 4 (m=4). A catalog de novo was created using the *cstacks* unit, which aligned the reads of a subset of 20 individuals from different geographical locations. The catalog was created with a subset of individuals because the extremely high number of different loci sequenced in all the individuals led to a crash of the program when aligning the sequences (> 3 million loci with 50 individuals analyzed). Individual reads were then aligned on the catalog with the *sstacks* unit, and the *population* unit was used to select loci with an individual coverage higher than 5 (parameter *m*). The output containing individual genotypes has been filtered using the VCFtools software (Danecek *et al.*, 2011) to remove the SNPs with a mean coverage below 8 or above 50. We selected the 250 individuals best sequenced (*i.e.* with the lowest rate of missing data), and removed the SNPs with more than 30% of missing data. Only 50 SNPs passed these filters, which supported the necessity to group individual in pools to infer reliable genotypes for an acceptable number of SNPs. The filters used for SNPs calling are summarized in Table S2.

**References**

Bradbury PJ, Zhang Z, Kroon DE, Casstevens TM, Ramdoss Y, Buckler ES (2007). TASSEL: software for association mapping of complex traits in diverse samples. *Bioinformatics* **23**: 2633–2635.

Catchen JM, Amores A, Hohenlohe P, Cresko W, Postlethwait JH (2011). Stacks: Building and Genotyping Loci De Novo From Short-Read Sequences. *G3 Genes Genomes Genet* **1**: 171–182.

Danecek P, Auton A, Abecasis G, Albers CA, Banks E, DePristo MA, *et al.* (2011). The variant call format and VCFtools. *Bioinformatics* **27**: 2156–2158.

Lu F, Lipka AE, Glaubitz J, Elshire R, Cherney JH, Casler MD, *et al.* (2013). Switchgrass Genomic Diversity, Ploidy, and Evolution: Novel Insights from a Network-Based SNP Discovery Protocol. *PLOS Genet* **9**: e1003215.

Normandeau E (2016). *Command line Stacks Workflow*.

**Supporting Figures**

**
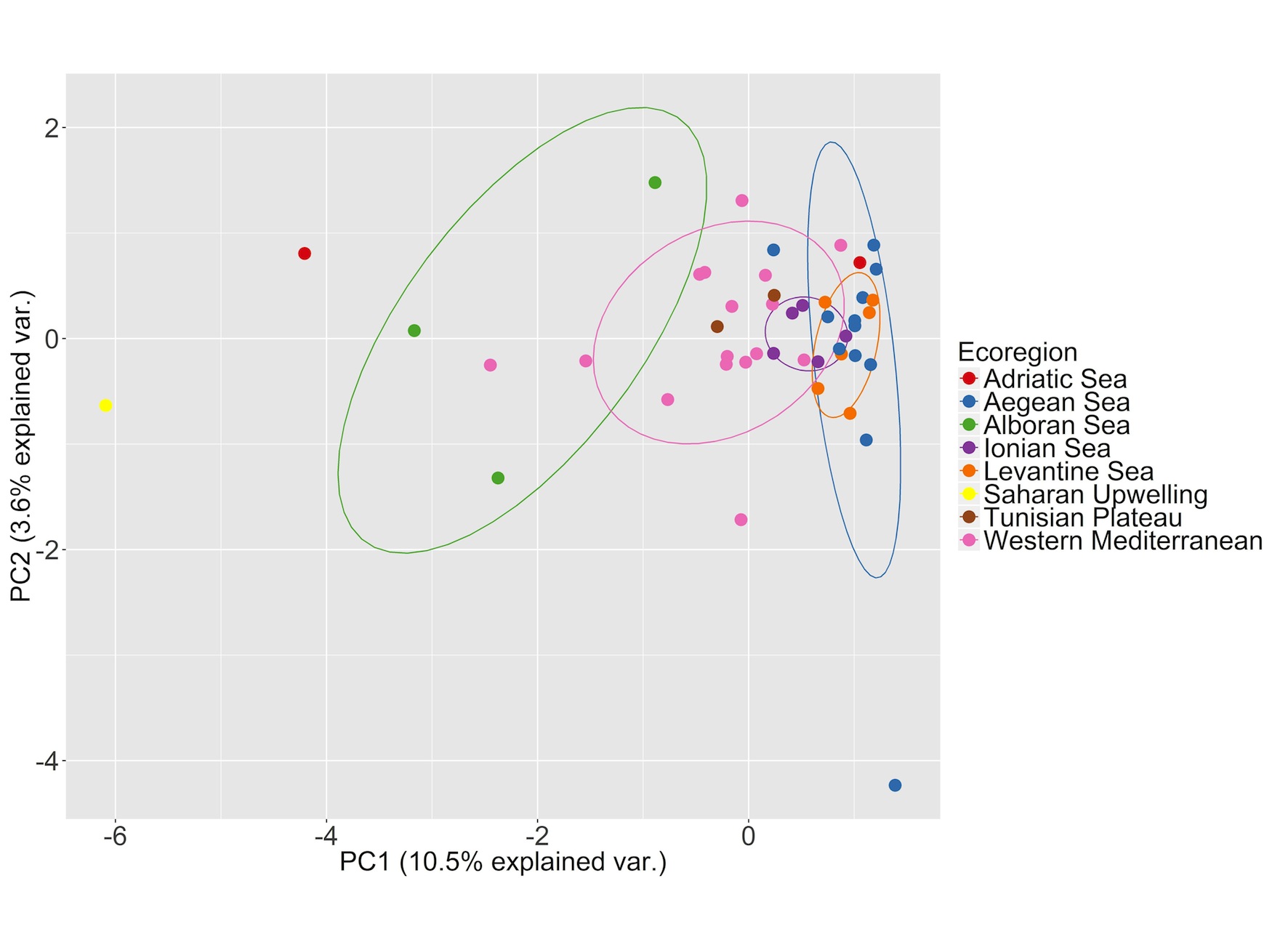
**

**Figure S1:** Principal coordinates analysis (PCoA) of the 47 sites computed using the Nei genetic distance calculated from SNPs allele frequencies. The colors and the circles indicate the ecoregions each site belongs.


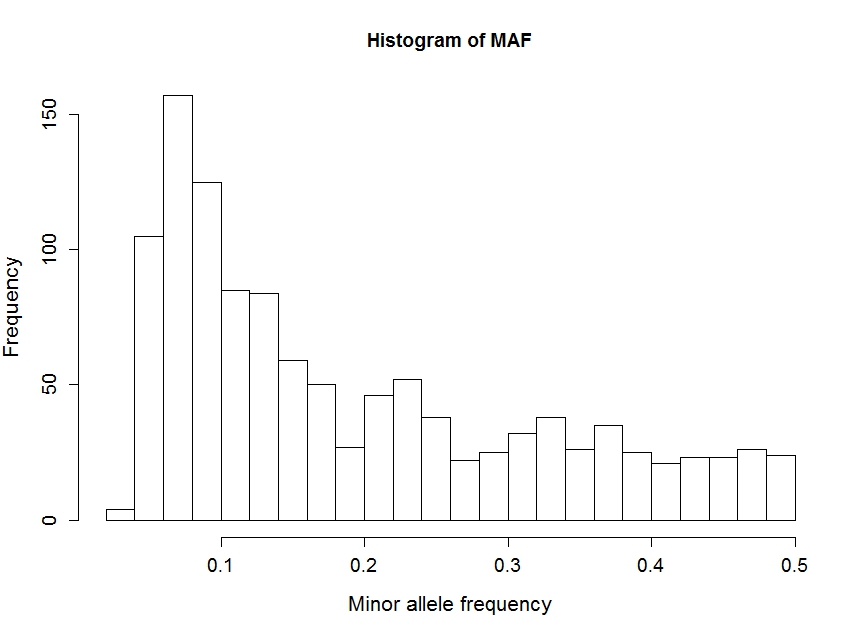


**Figure S2:** Histogram showing the distribution of minor allele frequency per markers (1153 SNPs) for the 47 sites.

**Supporting Tables**

**Table S1**. Number of raw reads and filtering data for each sequenced library. The percentage of filtered data indicates the proportion of sequences that was kept in the UNEAK analysis.

| **Library name** | **# raw sequences** | **# filtered sequences** | **%**  **filtered** |
| --- | --- | --- | --- |
| raw_C6JATANXX_1_fastq.txt | 293626046 | 152365277 | 51.89 |
| raw_C6JATANXX_2_fastq.txt | 265698080 | 250811438 | 94.40 |
| raw_C6JATANXX_3_fastq.txt | 272001986 | 254706376 | 93.64 |
| raw_C6JATANXX_4_fastq.txt | 264688559 | 206020993 | 77.84 |
| raw_C6JATANXX_5_fastq.txt | 292012357 | 271356748 | 92.93 |
| raw_C6JATANXX_6_fastq.txt | 279153586 | 255075707 | 91.37 |
|  |  | Average = | 83.68 |

**Table S2.** Parameters used to filter data in the SNPs calling procedure using UNEAK and Stacks.

| **Parameters** | **UNEAK** | **Stacks** |
| --- | --- | --- |
| Read length (bp) | 64 | 80 |
| Maximum number of mismatch in tags | 1 | 3 |
| Minimal coverage for tags alignment | 5 | 4 |
| Minimal coverage for alignment on the catalog | - | 5 |
| **Individual filters** | | |
| Minimal mean coverage per locus | 5 | 8 |
| Maximal mean coverage per locus | 10 | 50 |
| Minimal allele frequency | 0.05 | 0.05 |
| Maximal percentage of missing data per locus | - | 30% |
| **Pool filters** | | |
| Minimal coverage per locus per pool | 10 | - |
| Maximal percentage of missing data per locus | 0% | - |

**Table S3**. Ecoregion, description, site number, location, number of adults of *Mullus surmuletus* sampled (*n*) and expected heterozygosity (H_S_) of the 47 sampling sites used in the analyses.

| **Site** | **Site #** | **Country** | **Latitude** | **Longitude** | ***n*** | **Hs** |
| --- | --- | --- | --- | --- | --- | --- |
| Gibraltar | 20 | Spain | 36.0155 | -5.2157 | 10 | 0.27 |
| Santa pola | 3 | Spain | 38.1684 | -0.1918 | 9 | 0.261 |
| Marbella | 4 | Spain | 36.5990 | -4.0531 | 9 | 0.263 |
| Aguilas | 5 | Spain | 36.8924 | -1.9338 | 9 | 0.268 |
| Denia | 45 | Spain | 39.8159 | 0.3926 | 10 | 0.259 |
| Torredembara | 46 | Spain | 40.9500 | 2.0600 | 10 | 0.26 |
| Menorca | 100 | Spain (Balearic) | 39.8701 | 3.9435 | 9 | 0.258 |
| East Mallorca | 24 | Spain (Balearic) | 39.4800 | 3.5300 | 10 | 0.258 |
| Ibiza | 68 | Spain (Balearic) | 38.8311 | 1.3928 | 9 | 0.264 |
| West Mallorca | 99 | Spain (Balearic) | 39.4056 | 2.5751 | 9 | 0.271 |
| Marseille | 48 | France | 43.1260 | 4.7681 | 10 | 0.248 |
| Bonifacio | 14 | France (Corsica) | 41.2288 | 9.5491 | 10 | 0.26 |
| Cala Gonone | 16 | Italy (Sardinia) | 40.3453 | 9.8254 | 10 | 0.261 |
| North Corsica | 19_27 | France (Corsica) | 42.9300 | 9.1700 | 17 | 0.268 |
| East Sardinia | 13_15 | Italy (Sardinia) | 41.0100 | 8.1600 | 17 | 0.268 |
| Cagliari | 18 | Italy (Sardinia) | 39.1072 | 9.3990 | 9 | 0.26 |
| Tropea | 51 | Italy | 39.3350 | 15.7479 | 9 | 0.255 |
| Capri | 52_53 | Italy | 41.2109 | 13.0726 | 16 | 0.261 |
| Annaba | 88 | Algeria | 37.2780 | 7.2594 | 10 | 0.264 |
| Bizerte | 89 | Tunisia | 37.3368 | 9.0391 | 10 | 0.257 |
| Tunis | 90_92 | Tunisia | 35.6302 | 11.1504 | 17 | 0.267 |
| Zarsis | 91 | Tunisia | 33.3467 | 12.1275 | 10 | 0.258 |
| Crotone | 25_84 | Italy | 38.5500 | 16.7700 | 11 | 0.256 |
| Gallipoli | 85 | Italy | 40.0263 | 17.2276 | 9 | 0.248 |
| South Sicily | 12_87 | Italy (Sicily) | 36.8100 | 15.2600 | 15 | 0.261 |
| West Sicily | 11_86 | Italy (Sicily) | 37.7800 | 12.3200 | 18 | 0.26 |
| Kefalonia | 32_43 | Greece | 36.9400 | 21.5300 | 11 | 0.262 |
| Monfalcone | 80_82 | Italy | 43.6570 | 14.6014 | 14 | 0.261 |
| Monopoli | 83 | Italy | 40.6367 | 18.0343 | 10 | 0.252 |
| North Crete | 29_96 | Greece | 35.2844 | 25.7697 | 17 | 0.266 |
| Keramoti | 30 | Greece | 40.6046 | 24.4465 | 9 | 0.248 |
| Pereas | 34 | Greece | 40.0470 | 22.8903 | 10 | 0.253 |
| Naxos | 35 | Greece | 36.7800 | 25.5800 | 9 | 0.255 |
| Galatas | 36_8 | Greece | 37.8249 | 24.1212 | 12 | 0.263 |
| Sifnos | 37 | Greece | 37.2232 | 24.6743 | 10 | 0.253 |
| Skyros | 38_39 | Greece | 39.1457 | 23.9894 | 11 | 0.258 |
| Karpathos | 40_41 | Greece | 36.3271 | 27.4313 | 14 | 0.257 |
| Pyrgadikia | 9 | Greece | 40.0641 | 23.5063 | 18 | 0.245 |
| South Crete | 95 | Greece | 34.7800 | 25.2167 | 10 | 0.258 |
| Cesme | 6 | Turkey | 38.5700 | 26.1900 | 9 | 0.25 |
| Assos | 33_69 | Turkey | 39.6117 | 26.0826 | 13 | 0.258 |
| Gazipasha | 55_56 | Turkey | 35.9400 | 33.2368 | 18 | 0.259 |
| Samandag | 72 | Turkey | 36.2235 | 35.6724 | 10 | 0.256 |
| Kumyali | 10 | Cyprus | 35.3433 | 34.4483 | 10 | 0.257 |
| West Cyprus | 93_94 | Cyprus | 34.7800 | 32.3000 | 16 | 0.263 |
| Acre | 73 | Israel | 33.7650 | 35.1200 | 9 | 0.258 |
| Jaffa | 74 | Israel | 31.6697 | 34.0439 | 9 | 0.247 |
